# Stable Superhydrophobic and Antimicrobial ZnO/Polytetrafluoroethylene Films via Radio Frequency (RF) Magnetron Sputtering

**DOI:** 10.3390/mi14071292

**Published:** 2023-06-24

**Authors:** Aoyun Zhuang, Ke Wu, Yao Lu, Jianping Yu

**Affiliations:** 1 Key Laboratory of Air-Driven Equipment Technology of Zhejiang Province, College of Mechanical Engineering, Quzhou University, Quzhou 324000, China; yujianping@zju.edu.cn; 2 Department of Chemistry, University College London, 20 Gordon Street, London WC1E 0AJ, UK; ke.wu.12@ucl.ac.uk; 3 Department of Chemistry, School of Physical and Chemical Sciences, Queen Mary University of London, London E1 4NS, UK; yao.lu@qmul.ac.uk

**Keywords:** superhydrophobic, antimicrobial, ZnO/PTFE, RF magnetron sputtering

## Abstract

In this study, superhydrophobic ZnO/Polytetrafluoroethylene (ZnO/PTFE) films with water droplet contact angles (CA) observed as high as 165° and water droplet sliding angles of (SA) <1° have been prepared on glass substrates by RF magnetron sputtering. The PTFE was wrapped on a nano-rod made of a ZnO film with superhydrophobic properties while providing excellent UV resistance compared to hexadecyltrimethoxysilane (HDTMS) hydrophobic agents. The upper surface of the rough ZnO film was coated with PTFE, and most of the underlying coating was bare ZnO, which could well make contact with bacteria. For the Gram-negative strain, *E. coli*, the cell viability count of the ZnO/PTFE sample (3.5 log reduction, 99.96%) was conspicuously lower than that of the ZnO/HDTMS sample (1.2 log reduction, 93.87%) under 1 h illumination of UV light, which showed that the ZnO/PTFE sample has a better photocatalytic property than the ZnO/ HDTMS films. The ZnO/PTFE films also showed good mechanical robustness, which is an important consideration in their widespread real-world adoption.

## 1. Introduction

Bacterial biofilms are known as a serious threat to human health which can cause secondary contamination in their transportation, storage, sales and use [1]. Bacterial infections lead to the deaths of a large number of patients worldwide every year [2,3,4]. The use of antibacterial products is considered an effective way to prevent microbial harm, which can reduce people’s chances of disease and medical expenses [5,6].

At present, there are two main research points for antibacterial materials; one is to inhibit the formation of bacterial biofilms on the surface, and the other is to kill the bacteria present on the coating [7,8,9]. Superhydrophobic surfaces (SHS) showing low bacterial adhesion due to their self-cleaning performance have been considered a promising strategy to limit bacterial attachment and subsequent biofilm formation [10,11,12,13]. There are many methods for preparing superhydrophobic coatings reported in recent years, which are mainly divided into two types: one is to change the roughness to obtain superhydrophobicity, and the other is to reduce the surface energy through chemical modification to form a superhydrophobic surface. In our earlier work, nano-structured ZnO/HDTMS coatings with excellent superhydrophobic properties have been successfully prepared by radio frequency magnetron sputtering, which has the advantages of convenience, good controllability of parameters, and easy mass production. However, Hwang et al. [14] showed that the anti-adhesion activity of superhydrophobic surfaces is short-lived and that their rough nature may actually enhance bacterial colonization over the longer term, which is bad for their use in healthcare or food-preparation environments.

The photocatalytic method is widely used in the field of sterilization due to its environmental protection and high efficiency [15,16,17]. Some metal elements such as silver, titanium, and zinc can absorb ultraviolet light to activate oxygen in the air or water to produce hydroxyl radicals and reactive oxygen ions that react with bacterial cells, destroying their normal structure and thereby causing them to die or lose their ability to proliferate [18,19,20,21]. The coatings, when combined with superhydrophobic and photocatalytic properties, can effectively reduce bacterial adhesion and kill adherent bacteria, but the hydrophobic agent could be simultaneously decomposed under photocatalysis. This has also been verified in this paper, and the hydrophobicity of ZnO/HDTMS decreased significantly after a few hours of exposure to UV light.

In previous research reports, almost all micro-/nano-structured surfaces of superhydrophobic antibacterial coating samples have a low surface energy. Few have prepared superhydrophobic coatings with low surface energy on the upper surface and superhydrophilic properties inside the coating, or studied the antibacterial properties of the coatings. Since RF magnetron sputtering does not require the target as an electrode to be conductive, DC magnetron sputtering is limited to the use of metal targets or non-metallic targets with a resistivity within a certain range. Therefore, the RF magnetron sputtering method is chosen to prepare the PTFE thin film. In this paper, ZnO, due to its photocatalytic property, was used to construct the rough rod-like nanostructure of the superhydrophobic coating based on RF magnetron sputtering, and PTFE, due to its low surface energy (~18 mN/m) and excellent UV resistance, was coated on the nanorods on the upper surface of the rough ZnO coating so that the entire ZnO/PTFE coating had excellent superhydrophobic properties. The vast majority of the underlying coating is still ZnO, which contributed to it maintaining its own photocatalytic properties.

## 2. Materials and Methods

### 2.1. Materials

The Zn targets (99.99% purity, diameter of 60 mm, and thickness of 5 mm) and the PTFE targets (diameter of 60 mm and thickness of 5 mm) were purchased from Beijing HeZong Science & Technology Co., Ltd., Beijing, China, absolute ethanol (99.5%) was obtained from Chengdu KeLong Chemical Co., Ltd., Chengdu, China, deionized water (15.6 MΩ·cm) was used to clean the substrates. Standard microscope glass slides and the sand grains that were used to test the wear resistance were purchased from VWR International, Inc., Radnall, PA, USA.

### 2.2. Fabrication

Microscope glass slides were ultrasonically cleaned in absolute ethanol and deionized water for 10 min in each and dried for 30 min in a drying oven at 90 °C before use as substrates. Zinc coatings were prepared by RF magnetron sputtering (JPGF-480, Shenyang Scientific Instruments Co., Ltd., Shenyang, China) of a Zn target. The glass slides were fixed in the deposition chamber at a distance of 10 cm from the target. The chamber was pumped to vacuum (5 × 10^−3^ Pa) before introducing argon gas. Films were deposited with a constant sputtering power (120 W) under Ar atmosphere at 1 Pa for 15 min. The deposited films were then annealed to the ZnO film in a muffle furnace at 400 °C for 30 min. Upon cooling to ambient temperature, the ZnO films were fixed in the deposition chamber at a distance of 10 cm from the PTFE target. The chamber was pumped to vacuum (5 × 10^−3^ Pa) before introducing argon gas. The ZnO films were coated for 2 min with PTFE using RF magnetron sputtering (120 W, 1 Pa), and finally yielding superhydrophobic ZnO/PTFE surfaces. The process for the fabrication of ZnO/HDTMS sample can be found in our previous work [22].

### 2.3. Characterization

The wettability of the samples was evaluated using an optical contact angle meter (Drop Meter A-100P, MAIST Vision Inspection & Measurement Co., Ltd., Ningbo, China). The surface morphology and the elemental composition of the superhydrophobic samples were observed by a field emission scanning electron microscope (FESEM/EDS, S-3400N, Hitachi Ltd., Tokyo, Japan) equipped with energy dispersive X-ray spectroscopy (EDS), for which samples were prepared by sputtering a thin layer of Au onto the surface. ATR-FTIR measurements were taken over a range of 700–4000 cm^−1^ using a Perkin-Elmer Spectrum-100 (Ge crystal) equipped with a universal ATR attachment.

### 2.4. UV Activated Antimicrobial Test

Both the Gram-positive and Gram-negative bacteria were used to assess the antimicrobial activity of the material. The protocol was adapted from that of Macdonald et.al. [23]. For each test, one bacteria colony was incubated in brain heart infusion broth (BHI, Oxiod) at 37 °C with a shear speed of 200 rpm for 18 h. The pellet was recovered by centrifugation (5000 rpm for 5 min) and then washed with a sterilized phosphate-buffered saline (PBS, 10 mL) and re-suspended in PBS solution (10 mL). The washing process was repeated three times. The bacterial suspension was diluted to 1000-fold by putting the 10 µL aliquot into 10 mL of fresh PBS in order to obtain an initial inoculum with approx. 105~106 CFU/mL. A total of 15 µL of the initial inoculum was placed on top of each specimen and covered with a sterile cover slip (22 mm × 22 mm) (VWR). The specimen was then irradiated by UV light (UVItec LI-208.BL, 2 × 8 W, 365 nm, ~0.16 mW/cm^2^) for up to an hour. A further set of samples was maintained in the dark for the same time period as the UV irradiation. Post irradiation, each sample system was added to sterilized PBS (450 µL) and vortexed (30 s). The neat suspension (450 µL) was diluted in a stepwise process up to 100-fold. Each ten-fold serial dilution (100 µL) was placed onto an appropriate agar (Macconkey agar for *E. coli* and Manitol Salt agar for *S. aureus*) for viable counts. The plates were incubated aerobically at 37 °C for 24 h (*E. coli*) or 48 h (*S. aureus*). Each sample type contained two technical replicates and each test was reproduced three times.

## 3. Results

### 3.1. Chemical Composition

**Figure 1:**
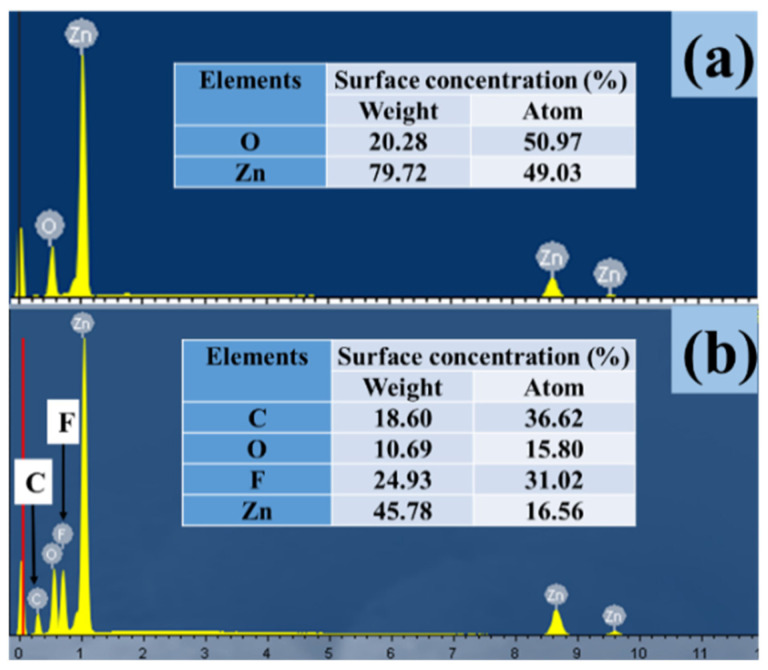
EDS spectra of ZnO film (**a**) and ZnO/PTFE film (**b**) of the superhydrophobic ZnO/PTFE film.

Thin films of ZnO/PTFE were deposited on microscope slides via RF magnetron sputtering with Zn and PTFE targets. The films covered 100% of the substrate and were well adhered to the glass. Figure 1a,b show the EDS spectra of ZnO and the ZnO/PTFE film; the atom concentrations of Zn and O elements on the ZnO film were 50.97% and 49.03%, respectively. The elemental composition of the superhydrophobic ZnO/PTFE film appears to have Zn, O, C and F, with the atom concentrations corresponding to 16.56%, 15.80%, 36.62% and 31.02%, respectively.

### 3.2. SEM Analysis

**Figure 2:**
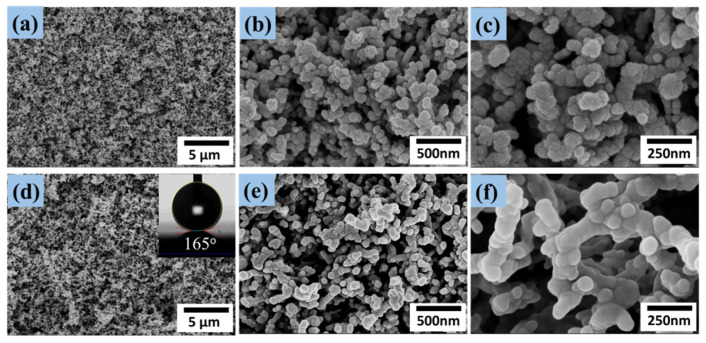
FE-SEM images of the superhydrophilic ZnO film (**a**–**c**) and the superhydrophobic ZnO/PTFE film (**d**–**f**), and an optical photograph of the water droplet on the superhydrophobic ZnO/PTFE surface.

Superhydrophobic ZnO/PTFE thin films were successfully deposited by RF magnetron sputtering whereby textured ZnO was sputtered onto the glass substrate, followed by a coating of PTFE to yield the superhydrophobic films. Scanning electron microscopy (SEM) of the films revealed a highly textured nanostructure at various magnifications, as shown in Figure 2. It is apparent that the untreated ZnO surface uniformly comprised popcorn-like clusters of nanoparticles, as shown in Figure 2a–c. The porous structure suggests a large surface area available for coating by the PTFE, as well as great potential for the trapping of air by sessile water droplets; both are important factors in the fabrication of superhydrophobic surfaces. Figure 2d–f show the structure of the ZnO surface coated with PTFE sputtered for 2 min under 100 W. It was found that the diameter of the nanoparticles in Figure 2f were a little larger compared with those in Figure 2c due to the thin PTFE film coating, which turned the superhydrophilic ZnO surface into the superhydrophobic ZnO/PTFE surface.

To describe the relationship between surface wettability and heterogeneous surfaces, Cassie and Baxter [24,25] proposed the following equation:

cos *θ_c_* = cos *θ* − *f*_2_ (cos *θ* + 1), (1)

where *f*_2_ is the fraction of air in the composite surface, and *θ_c_* and *θ* are the contact angles on the rough and untextured surfaces, respectively. When the contact angle *θ* of the untextured surface is constant, a lower *f*_2_ would lead to a larger contact angle *θ_c_* of the superhydrophobic surface. The contact angle of the surface increased to 165°, as shown in Figure 2d, which is far above that of the bare surface, and the sliding angle greatly reduced to less than 1° demonstrating superhydrophobic properties. The contact angle of the flat PTFE film has been shown to be 116°. The *f*_2_ value was calculated for the superhydrophobic ZnO/PTFE film to be about 0.94 according to Equation (1), which indicates that the actual fraction of contact area between the solid surface and the water surface was only 0.06.

### 3.3. UV Resistance

**Figure 3:**
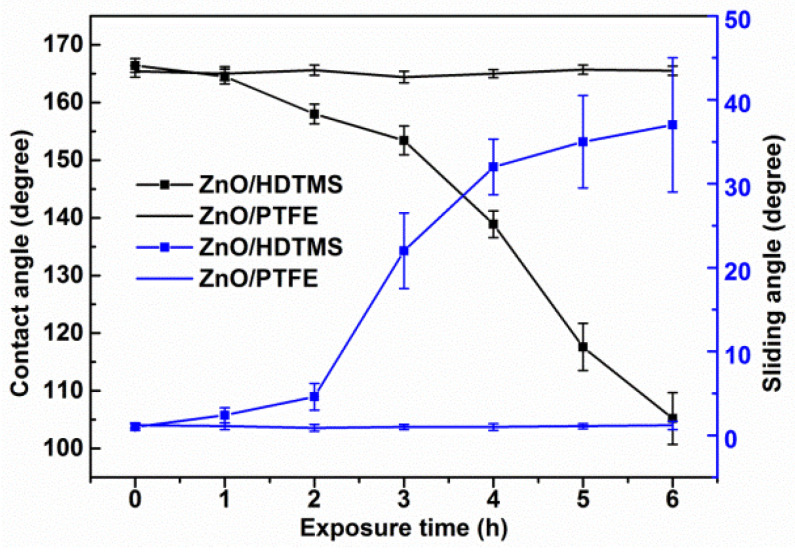
Contact angles and sliding angles of the superhydrophobic ZnO/HDTMS and ZnO/PTFE films after different UV exposure times (320–420 nm, 0.9 W/m^2^, 25 °C).

In order to test the UV resistance of the superhydrophobic ZnO/PTFE surfaces, the samples were exposed to UV light (320–420 nm, 0.9 W/m^2^) for 6 h at 25 °C, and the contact angle (CA) and sliding angle (SA) of the ZnO/PTFE and ZnO/HDTMS surfaces were both measured after each 1-h period, as shown in Figure 3. It was found that the wettability of ZnO/HDTMS surfaces dropped significantly after irradiation, with the CA decreasing from 166° to 105° and the SA increasing from 1° to 37°. Meanwhile, the ZnO/PTFE surface still exhibited consistent superhydrophobicity with a contact angle of 165° and a sliding angle < 1° after irradiation for 6 h, showing a superior UV-stability. This can be ascribed to the C–F bonds with high bond energy (485 kJ/mol) on the long chain of PTFE coated on to the ZnO surface. These C–F bonds cannot be broken by the UV light (314–419 kJ/mol) [26]. But, the C–H bonds of HDTMS could be easily damaged by the UV light.

### 3.4. Antimicrobial Performance

The antimicrobial efficacy of the ZnO/PTFE and ZnO/HDTMS samples were quantitatively assessed by adopting a well-developed plate count method under UV illumination. Strains chosen were spread across Gram-positive bacterium and Gram-negative bacterium. Staphylococcus aureus 8325-4 [27] is one of the representative staphylococcal lineages that was refined for laboratory use. E. coli ATCC 25922 [28], a non-diarrheagenic pathogen that is considered as standard, is a commonly used clinical strain for microbiological research.

**Figure 4:**
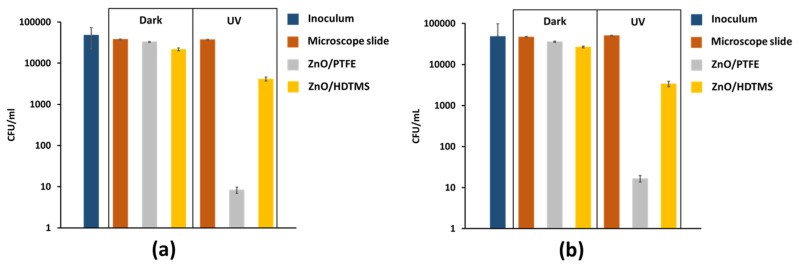
Antimicrobial assay illustrating the kill activity induced by the microscope slide and treated superhydrophobic microscope slides when tested against (**a**) *S. aureus* and (**b**) *E. coli* at 23 °C under UV irradiation and in the dark for 30 min and 1 h, respectively. Light source emitted with an average intensity of 0.16 mW/cm^2^. Error bars are standard deviation.

In this study, we investigated the CFU counts on the ZnO/PTFE and ZnO/HDTMS superhydrophobic surfaces and their corresponding controls in response to UV light with mild intensity. Each strain was used in a total of three trials so as to obtain reliable and reproducible results. Figure 4a,b show that upon irradiation with UV light, both *S. aureus* and *E. coli* bacteria were growing on the bare microscope slides. This indicates that the long wavelength UV light on its own is not capable of eradicating either of the bacterium. The CFU counts of bare microscope slides in both dark and illuminated conditions also suggest that the slides provide a hospitable “hotbed” for bacteria to proliferate on at room temperature.

From Figure 4a,b the graphs reveal the same trend with neither the ZnO/PTFE nor the ZnO/HDTMS superhydrophobic samples exhibiting significant antibacterial activity under dark conditions when tested against *S. aureus* and *E. coli*. The ZnO/PTFE sample has 0.06 log (13%) and 0.12 log (24%), respectively, and ZnO/HDTMS sample has 0.24 log (~43%) for both strains compared to the dark control (microscope slide). It is postulated that the trivial bactericidal activity of ZnO in both the ZnO/PTFE and ZnO/HDTMS samples in the absence of a light source can be attributed to the attachment of ZnO to bacterial cell walls, which subsequently causes the dissolution of local ZnO and therefore increases the concentration of Zn^2+^ ions within the bacterial cytoplasm [29].

**Figure 5:**
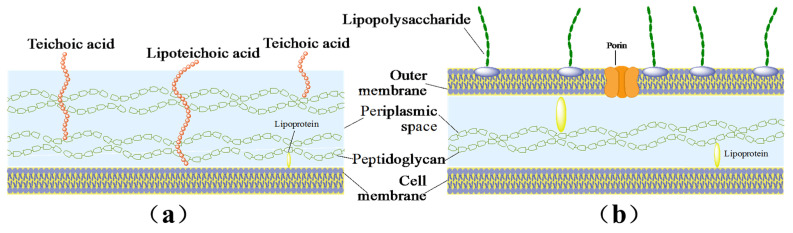
Cell wall structures of (**a**) Gram-positive bacteria and (**b**) Gram-negative bacteria.

Both the teichoic acid and lipopolysaccharide shown in Figure 5a,b are rich in polyphosphate anions in these two species, and can be found in peptidoglycan layer of the Gram-positive bacteria and the outer membrane of the Gram-negative bacteria, respectively. They are considered to be the active sites for ZnO to attach to the bacteria cell wall [30,31]. As a consequence, teichoic acid or lipoteichoic acid could facilitate ZnO dissolution via the formation of ionic salts with Zn^2+^ ions. The Zn^2+^ ions then reach the cytoplasm through the peptidoglycan layer or outer membrane via the facilitator metalloproteins, and therefore becomes cytotoxic [32].

With long wavelength irradiation (365 nm), however, both the ZnO/PTFE and ZnO/HDTMS surfaces demonstrated remarkable antimicrobial activity. Illumination of samples containing ZnO resulted in the enhancement of photo-activated bactericidal activity. The ZnO/PTFE sample obtained a 3.7 log reduction when tested against *S. aureus* whereas the ZnO/HDTMS sample demonstrated a 0.9 log reduction for a 30 min incubation duration, which are equivalent to 99.98% and 88.86% reductions compared to their illuminated control. For the Gram-negative strain, *E. coli*, the cell viability count of the ZnO/PTFE sample (3.5 log reduction, 99.96%) was conspicuously lower than that of the ZnO/HDTMS sample (1.2 log reduction, 93.87%) under 1 h illumination of UV light. Both of these results show that the ZnO/PTFE sample has a greater photobactericidal property than the ZnO/HDTMS sample.

ZnO is believed to have an intrinsic photocatalytic efficiency; therefore, it can absorb UV radiation efficiently [33]. This characteristic enables the ZnO to interact with the bacteria. Upon UV illumination, any loosely attached oxygen will desorb from the surfaces and therefore be converted to a reactive oxygen species (ROS) such as H_2_O_2_ OH^−^ and O^2−^. Therefore, by penetrating the bacterial cell, these active species can eradicate microorganisms. Compared with the ZnO/HDTMS sample, in which ZnO nanorods were completely coated with the modifier, only the upper part of the ZnO/PTFE sample is coated in PTFE; the entire underlying coating was bare ZnO, which could well make contact with bacteria, showing its photocatalytic property.

### 3.5. Wear Resistance

**Figure 6:**
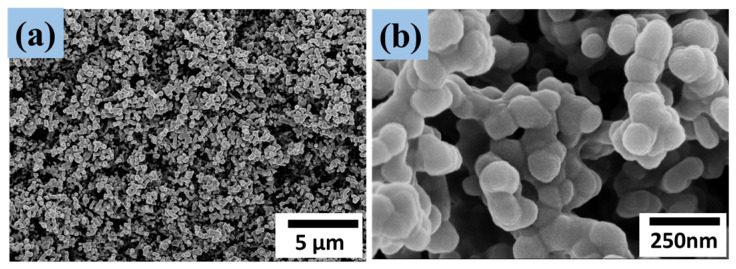
SEM images (**a**,**b**) of the superhydrophobic ZnO/PTFE film at different magnifications after the sand impingement from 50 cm height.

To test the wear resistance of the ZnO/PTFE films, 10 g of sand grains was dropped from a 50 cm height onto the 30° tilted ZnO/PTFE surface, as shown in the online supplementary Video S1. After the impact of the sand grains, the water droplet CA and SA were measured; the CA was still higher than 153° and the SA was <5°, and thus retained excellent superhydrophobicity. Figure 6a,b shows the surface structure of the superhydrophobic ZnO/PTFE film after the sand impingement. As can be seen from the figure, the nano-sized ZnO/PTFE protrusions on the film surface still maintained a good roughness. The same method was used to test the wear resistance of the ZnO/HDTMS films, and the results showed that the contact angle was still greater than 152° and the sliding angle was less than 5°. This observation is apparently due to the strong adhesion of the ZnO clusters to the glass substrate and the fact that most of the ZnO/PTFE protrusions were able to resist sand grain impingement at a certain height. This property is of great importance for the long-term use of this superhydrophobic film.

## 4. Conclusions

ZnO/PTFE films with nanorod structures were successfully fabricated on glass slides by RF magnetron sputtering using Zn and PTFE targets, which combined both superhydrophobic and bactericidal properties. The rod-like ZnO structure on the upper layer was wrapped by PTFE, which presented low surface energy, and thus demonstrated excellent superhydrophobic properties with water droplet contact angles of up to 165°, which can effectively resist the adhesion of bacteria. Moreover, most of the ZnO nano particles inside were in a bare state, showing superhydrophilic properties, which can remove the adhering bacteria by photocatalysis, achieving an excellent antibacterial effect. The ZnO/PTFE films also showed excellent stability for UV/wear resistance. This method for ZnO/PTFE deposition is cheap and straightforward, promising a route to larger-scale antimicrobial superhydrophobic coating fabrication, while the approach of combining superhydrophobic and superhydrophilic properties opens up the field to a huge variety of potential material combinations in antimicrobial coating designs.

## Data Availability

The data used to support the findings of this study are available from the corresponding author upon request.

## Supplementary Materials

The following supporting information can be downloaded at: https://www.mdpi.com/article/10.3390/mi14071292/s1, Video S1: Test for the wear resistance.
